# An Endpoint Detection System for Ion Beam Etching Using Optical Emission Spectroscopy

**DOI:** 10.3390/mi13020259

**Published:** 2022-02-05

**Authors:** Junjie Zhang, Jiahui Luo, Xudong Zou, Jiamin Chen

**Affiliations:** 1 State Key Laboratory of Transducer Technology, Aerospace Information Research Institute, Chinese Academy of Sciences, Beijing 100190, China; iecas_zjj@163.com (J.Z.); luojiahui18@mails.ucas.ac.cn (J.L.); zouxd@aircas.ac.cn (X.Z.); 2 School of Electronic, Electrical and Communication Engineering, University of Chinese Academy of Sciences, Beijing 100049, China

**Keywords:** etching endpoint detection, ion beam etching, optical emission spectroscopy

## Abstract

An ion beam etching system with etching endpoint detection (EPD) capability based on optical emission spectroscopy (OES) was conceived, built, and tested. An expansion chamber was added on the right side of the etching chamber to fix the optical detector for in-situ detecting. In this system, the optical detector was mounted on a seven-shaped bracket, which was fixed by two straight guides, thus the position of the optical detector could be adjusted arbitrarily to collect the emission spectrum generated by the sample during the etching process. The signal was transmitted by optical fiber to the computer for processing, and the etching endpoint could be detected after analyzing the data. Firstly, we used simple substances (Al, Cr, Si, and Mg) to analyze the feasibility of the system and determine the best position of the optical detector. In addition, we also tested the detection limit of the system. Finally, a complex multilayer film sample with different materials was tested, and the results showed that the system could clearly detect the characteristic emission lines of different layers and had a good real-time performance and excellent endpoint detection capabilities.

## 1. Introduction

Ion beam etching (IBE) is a dry etching process developed in the 1970s [1], which is well known for its high etching accuracy and wide range of etching materials. It has been widely used and has become an indispensable fabrication technology for micro/nano-electronic devices [2,3,4,5], especially in the field of GMR (giant magnetoresistance) and TMR (tunneling magnetoresistance) study, because it has no after-corrosion and oxidation problems [6,7]. However, as the size of thin-film devices continues to shrink, precise control of the etching process is required in many applications. At present, there are many etching endpoint monitoring methods in various etching systems, such as a Langmuir probe [8], mass spectrometer [9], laser, or plasma impedance monitoring [10]. However, these methods have some disadvantages; for example, the mass spectrometer method has a high cost, and, in the laser method, it is difficult to distinguish the films with similar etching rates.

On the other hand, optical emission spectroscopy (OES) is a very reliable and widely used analysis technique, especially for plasma analysis. OES is, nowadays, among the commonly used devices in plasma etching systems for etch endpoint detection, with successful use in the monitoring of the etching of complex devices or plasma chamber cleaning [11,12,13,14,15], and equipment manufacturers provide such EPD systems. Besides, the use of OES to monitor the etching in an IBE system is not frequent, although it has been reported before [16]. However, the EPD system above detects the endpoint through the chamber viewport window rather than in-situ, and this causes problems, such as signal attenuation because of the coating on the window by the sputtered materials. Therefore, high accuracy detection of etching endpoint for micro/nano-electronic devices is still challenging.

In this paper, we report a low-cost, in-situ, ion beam etching endpoint detection system based on optical emission spectroscopy and present the structure and performance test of the system. Emission spectroscopy is obtained by a spectrometer with a resolution of 0.8 nm. We expanded a chamber based on the original LKJ-1A-150 ion beam etcher to fix the detector for in-situ detecting of the emission spectrum: the design of the slide rail made the position of the detector adjustable so that it could detect the emission spectrum of any area on the sample stage. The optical detector was connected to the external spectrometer through an optical fiber and vacuum connector, and the collected emission spectrum was sent to the computer, thus we could distinguish the etching endpoint by analyzing the characteristic emission line intensity. The design of the shutter was introduced to make the etching start or stop immediately, avoiding instability when the ion source started, and helped to accurately control the etching process. The manufacturing cost of the whole system was about EUR 10,000, which is about 15 times less than that of the endpoint detection system using SIMS (secondary ion mass spectroscopy) under the same detection limit. The experimental results show that the system has a detection limit of 1 nm and has good performance for endpoint detection of a complex thin-film structure, which can better detect the emission spectrum of devices with minor size, thus it is expected to become a versatile tool for endpoint detection.

## 2. System Design

### 2.1. Overall Structure

**Figure 1:**
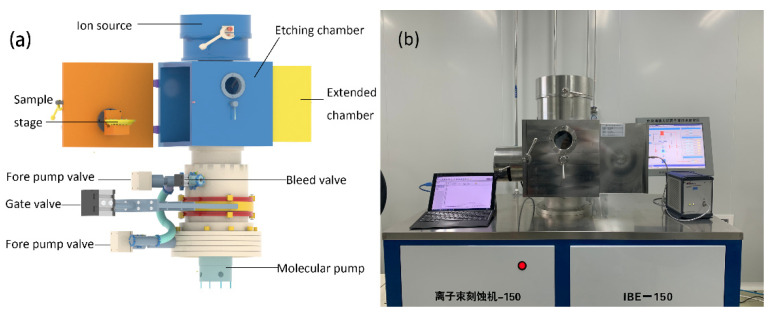
(**a**) The three-dimensional model of the system (**b**) The photograph of the system.

Figure 1a,b shows the three-dimensional (3D) model and photograph of the system, which is based on the LKJ-1A-150 Ion Beam Etcher. The overall system had two subsystems: one was the etching system above and the other was the air pump and water-cooling system below. The ion source in the etching chamber adopted a dual-gate structure and divergent field ion source. The radius (R) of the ion source was 150 mm, the maximum beam current ($I_{b}$) could reach 180 mA, the maximum discharge power ($P_{d}$) could reach 600 eV/Ion, the beam uniformity could reach 80% ± 5%, and the maximum beam density ($J_{b}$) was 1.8 mA/$cm^{2}$. The ion source produced plasma, and the plasma was introduced into the etching chamber after being accelerated by the accelerating grid electric field for etching samples.

A fore pump was installed under the etching chamber in order to make the chamber vacuum quickly. A molecular pump was installed under the fore pump and served as the main pump for the system; when the pressure of the etching chamber was below $10^{-3}\;\mathrm{pa}$, the molecular pump started working until the pressure met the demand. Cycles of ion bombardment could be performed in the etching chamber, and this process generated a lot of heat. Therefore, a water-cooling system was equipped for the sample stage, which could decrease the sample temperature by a continuous flow of liquid to prevent damages. Moreover, the optical detector was installed on the extended chamber for real-time monitoring; it could collect the emission spectrum during the etching process.

### 2.2. Expansion Chamber

**Figure 2:**
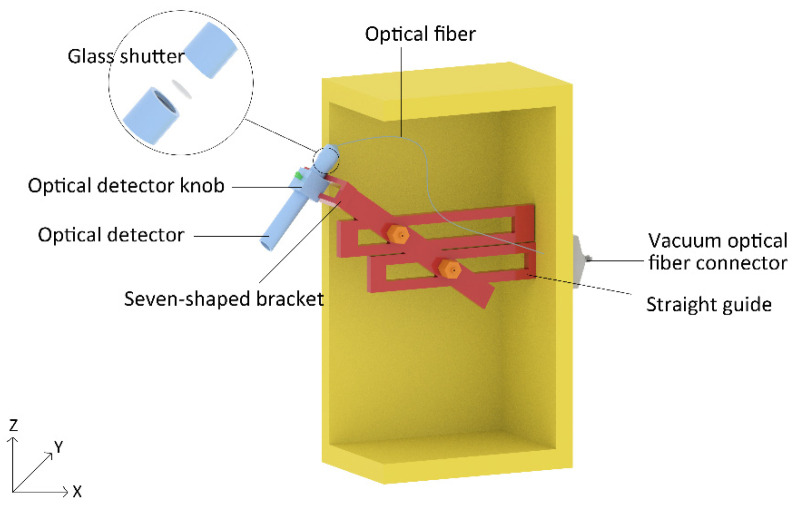
The internal model of the expansion chamber.

The expansion chamber used to place the optical detector was installed on the right side of the etching chamber for in-situ detecting. Compared with other designs that place the optical detector directly in the etching chamber, several advantages were expected: (1) the optical fiber damage could be avoided because it was hidden in the expansion chamber and the system could run for a long time; (2) the optical detector would block part of the ion beam, resulting in uneven etching; (3) the straight guide and the seven-shaped bracket in the expansion chamber could realize the three-dimensional movement of the optical detector so that it could collect the emission spectrum from any area in the sample stage. Besides, a vacuum optical fiber connector next to the expansion chamber was installed, which made sure the emission spectrum could transfer to the computer outside with transmission wavelengths from 200 nm to 940 nm while ensuring the vacuum environment of the vacuum chamber was not destroyed. The internal model is shown in Figure 2.

The optical detector was the most critical unit of the EPD system, as it largely determines the performance of the entire system. The internal design of the expansion part was mainly on the basis of the optical detector. The optical detector, which mainly included a collimating lens, shutter glass, and sleeve, was aligned with the surface of the etched sample to collect the light caused by ion bombardment. Then, the light was collected into the optical fiber through the collimating lens. However, the glass shutter and the collimating lens can degrade and contaminate over time. Furthermore, collimating lenses are more difficult to clean and maintain than shutter glass. Therefore, the function of the shutter glass was to block the etched deposits and maintain a stable light transmittance; the collimating lens was protected by changing the shutter glass frequently, and the replacement of the glass shutter was quite easy. As shown in Figure 2, the detector could be disassembled; we could replace the glass shutter as long as the detector was screwed apart. Optical fiber was used as a channel to transmit optical signals.

The optical detector was fixed by a seven-shaped bracket, two straight guides, and a detector knob; this modular design made it easy to assemble and maintain. On one rod of the seven-shaped bracket, there were two connection points to connect two straight guides, and on the other rod, there was a knob to connect with the optical detector. We used two screws at the connection points to realize the vertical (Z direction) and horizontal (X direction) movement of the optical detector relative to the two straight guides, and the knob made the optical detector move in the polar direction (Y direction). All three parts ensured that the optical detector collected the emission spectrum at any position on the sample stage, and the movable parts were installed in the expansion chamber and were subject to minor deposition, thus the deposition had little effect on them.

### 2.3. Shutter

**Figure 3:**
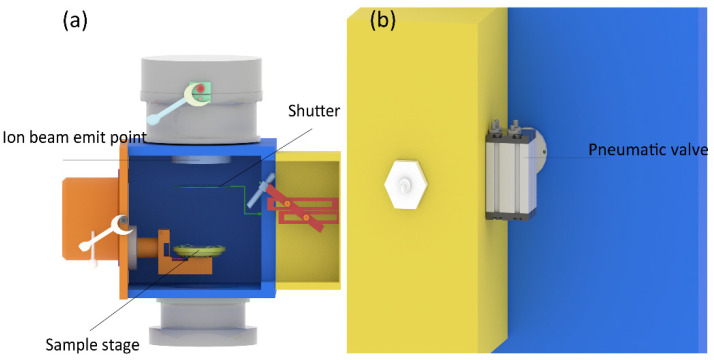
(**a**) The three-dimensional model and the position of the shutter, (**b**) the three-dimensional model and the position of the pneumatic valve.

The shutter was the key unit for precisely controlling the etching endpoint. It was placed in the etching chamber, as shown in Figure 3a. The lifting and lowering of the shutter plate were controlled by the external pneumatic valve, as shown in Figure 3b. When the shutter was lifted, the ion beam could be blocked, thus the etching of the sample could be stopped immediately. When the ion source power was turned off, the ion beam did not disappear instantly, it had a buffer time, and this buffer time caused the etching endpoint to be uncontrollable and severely damaged the sample structure, e.g., over-etching. It should be explained that the radius (R) of the shutter was 160 mm, which was larger than that of the ion source. Therefore, it was certain that the shutter could block almost all ion beams, and the design of the shutter could eliminate this buffer time, thus the etching endpoint could be precisely controlled.

## 3. Results

### 3.1. Simple Substance

In the conventional ion beam etching process, about 0.005% to 1% of the sputtered atoms could be in the excited state, while the general ion beam etching rate was about $10^{13}$ atoms/s, which means the OES method has a high sensitivity. To verify whether the spectrometer could detect the emission spectrum of the etching sample, we selected Al, Cr, Si, and Mg simple substances for experiments and detected the emission spectrum. The emission spectrum was obtained by a spectrometer, and its model was AvaSpec-HSC1024x58TEC-EVO. The spectrometer had a good resolution of 0.8 nm, and its slit width was 10 μm. Besides, the fiber cable was 600 μm core and SMA terminated fiber. All reported elements in this paper are clearly identified (spectroscopic notation, upper and lower energy levels, emitted wavelength); we selected the lines with the strongest intensity to list, as shown in Table 1.

**Figure 4:**
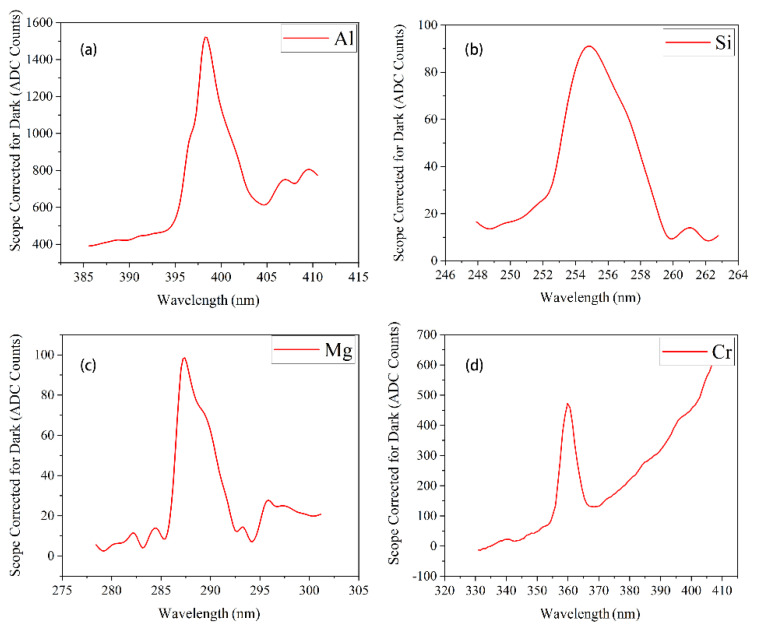
(**a**) The characteristic emission line spectra of Al (**b**) The characteristic emission line spectra of Si (**c**) The characteristic emission line spectra of Mg (**d**) The characteristic emission line spectra of Cr.

The results show that the spectrometer can accurately detect the spectral characteristic emission lines of different materials; meanwhile, the lines are obvious. Different materials had different characteristic emission lines, which could be used as the distinguishing criterion. The experimental results are shown in Figure 4.

Figure 4a is the emission spectrum of the Al simple substance; there is an obvious line around the wavelength of 398 nm. Compared with the database provided by NIST (National Institute of Standards and Technology), it was confirmed that the measured emission lines matched the theoretical emission lines of Al. Observing other metals, such as Si (b), Mg (c), or Cr (d), it was found that there were clearly distinguishable characteristic emission lines. Therefore, it can be concluded that the idea of using the emission spectrum to detect the etching endpoint is feasible in practice.

In addition, it needs to be explained that the emission lines presented in Figure 4a–d are broad and can cover several lines at different wavelengths. In Figure 4b, for example, there are several Mg’s emission lines between 284 nm and 286 nm. However, due to the limitation of spectrometer resolution, we combined these emission lines into an “emission line interval” and then set the spectral wave with the highest line intensity as the “main line”. Finally, we took the “main line” as the basis for monitoring the etching process (applied in the detection limit and application of Part IV).

### 3.2. The Setting of Optical Detector

Generally, the position of the optical detector will affect the emission spectrum. For example, the angle and distance between the optical detector and the sample will affect the determination of the etching endpoint to a certain extent, so the setting of the optical detector position is an important issue that needs to be considered in this design. In addition, it needs to be noted that this system was built to fabricate and study micro/nano-electronic devices with a complex multilayer film structure. The film thickness of these devices could reach 1 nm; therefore, the etching rate was the key factor affecting the yield of the devices. If the etching rate was too high, the emission spectrum of the thin film could not be detected (the etching time was less than the sampling time of the spectrometer). However, the etching rate could not be too low because this would result in the intensity of the emission spectrum being too small. The etching conditions (beam current = 95 mA, cathode current = 4.4 A, acceleration voltage = 200 V) we used were determined by many experiments. Under this condition, the emission spectrum of the film would not be difficult to detect, and the intensity would not be too small. Therefore, our experiments were carried out under this condition unchanged.

**Figure 5:**
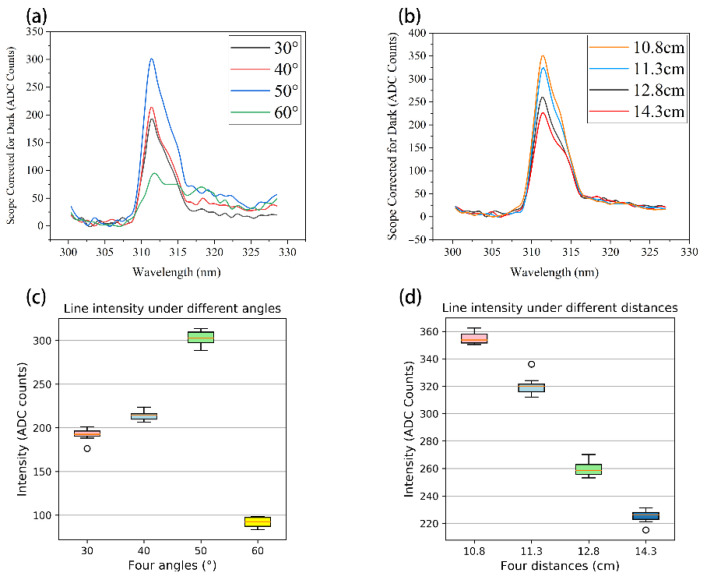
(**a**) The emission spectrum at different angles of 30°, 40°, 50°, and 60° (**b**) The emission spectrum at different distances of 11.3 cm, 12.8 cm, and 14.3 cm (**c**) The box plot of intensity at different angles (**d**) The box plot of intensity at different distances.

We first tested the system at different angles, ensuring that the optical detector was aligned with the center of the Al sample. Then, we kept the distance between the end of the optical detector and the sample at the same distance (12.8 cm) and adjusted the angle of the optical detector (angle with the sample stage) to obtain the emission spectrum at different angles. The experiment mainly tested the emission spectrum intensity at the angles of 30°, 40°, 50°, and 60°. Figure 5a shows the spectra at different angles (straight line subtracted). The quality of the emission spectrum was mainly determined by the intensity of the “main line”. The larger the intensity, the more accurately the lines could be recognized. This design selected 10 data for each angle to determine the optimal angle. Table 2 shows the measured data and the analysis results from the four angles. A box plot based on Table 2 is shown in Figure 5c.

From the above experimental results shown in Table 2, the emission spectrum intensity was strongest when the optical detector angle was 50°, and the intensity at other angles was relatively small. It was speculated that the photon energy released by sputtered atoms in the excited state was different along different angles, and the strongest angle matched 50° in this experiment. Therefore, the optical detector angle was set to 50° by the results of intensity.

In addition, we tested the system at different distances. We ensured that the optical detector was aligned with the center of the sample stage, keeping the optical detector angle at 50° unchanged, and obtained the emission spectrum at different distances. We selected the distance between the end of the optical detector and the sample to be 11.3 cm, 12.8 cm, and 14.3 cm to test and the intensity results are shown in Table 3. Figure 5b shows the spectrum at different distances. A box plot based on Table 3 is shown in Figure 5d.

According to the results above, there is a clear conclusion that the shorter the distance and the larger the intensity, the easier it is to distinguish the emission line. It is speculated that the light intensity produced by photon emission is mostly concentrated on the surface of the sample. Therefore, the distance between the optical detector and the sample should be as small as possible. However, in this design, the optical detector could not be infinitely close to the sample because if the optical detector extended out to the expansion chamber too long, some ion beams would be blocked, resulting in an uneven etching of the sample, and the optical detector would also be etched and damaged. We finally chose to set the distance between the optical detector and the sample to be the smallest without affecting the etching process; the measured distance was about 10.8 cm (the intensity was 355.09, as shown in Figure 5). In conclusion, the optimal angle and distance of the optical detector were 50 °and 10.8 cm, respectively.

## 4. Performance

### 4.1. Detection Limit

**Figure 6:**
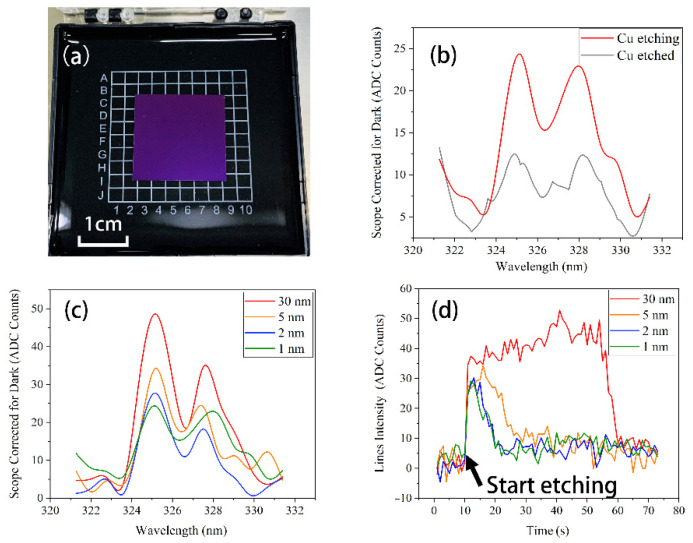
(**a**) Cu thin film samples with the size of 2 cm × 2 cm (**b**) The intensity comparison between Cu being etched and Cu having been etched (**c**) The emission line spectra of Cu thin film samples with different thickness (**d**) The changes over time in the intensity of the “main line” of Cu thin film samples with different thickness.

To test the detection limit of the whole system, a series of Cu samples (${\mathrm{SiO}}_{2}\;subs$) with different film thicknesses were used to characterize the system’s capability. The samples were obtained by depositing Cu films with different thicknesses on a SiO_2_ substrate and then dicing the samples to 2 cm × 2 cm, as shown in Figure 6a.

As previously stated, Cu has a “main line” between 321 nm and 331 nm; the wavelength of the “main line” is around 324.754 nm (the wavelength of 327.395 nm can also be regarded as a “main line”). We can detect the intensity changes of the “main line” to monitor the etching process of the Cu film. Figure 6b mainly demonstrates the line intensity changes between Cu being etched and Cu having been etched. The experiment mainly tested the Cu samples with 30 nm, 5 nm, 2 nm, and 1 nm.

Figure 6c shows the emission spectrum of the different samples when the Cu film is etched, which indicates that even the 1nm samples could be detected with the “main line”. We also found that the intensity was related to the film thickness, spanning from an intensity of 23.344 to 48.659. On the other hand, as shown in Figure 6d, changes over time in the intensity of the “main line” of different Cu samples could be achieved. When the etching process started, the intensity of the “main line” rose to a high level until the etching process ended. Meanwhile, the etching time extended with the increasing film thickness. Even the 1 nm sample had a good detection capability on the changes over time.

From the results of both the emission spectrum and the changes over time, it can be concluded that the system can detect a film change as thin as 1 nm, and the detection limit of the system can be 1 nm or thinner. This shows a higher accuracy and a better detection limit compared with other systems [14,17]. It also indicates a great advantage on endpoint detection for micro/nano-devices. 

### 4.2. Application

We built this system to fabricate and study devices with a complex multilayer film structure, like GMR and TMR devices. TMR and GMR magnetic sensors usually have a multilayer thin-film structure and have a high magnetoresistance ratio, and they have been widely used in recent years. In the manufacturing process of TMR and GMR devices, it is usually necessary to etch to the bottom layer (without over-etching) for electrode extraction, so the precise control of the etching process is a major issue. However, the OES-based endpoint detection system can monitor the etching process of multilayer devices, effectively prevent over-etching, and improve the yield of devices, which is very helpful for the fabrication of devices.

In order to explore the detection performance of the EPD system on the multilayer film with different materials, we tested a multilayer film sample (a TMR sample) to characterize. The film structure of the test sample was as follows: MgO sub.//Cr (40 nm)/Fe (100 nm)/Mg (Wedge 0–1 nm)/MgAl_2_O_4_ (Wedge 1–2.5 nm)/Fe (7 nm)/IrMn (12 nm)/Ru (10 nm). From left to right, different layers were stacked from bottom to top; MgO was the substrate. The etching energy was 550 eV, the beam current was 89 mA, and the spectrometer collected spectral data every 3 s.

**Figure 7:**
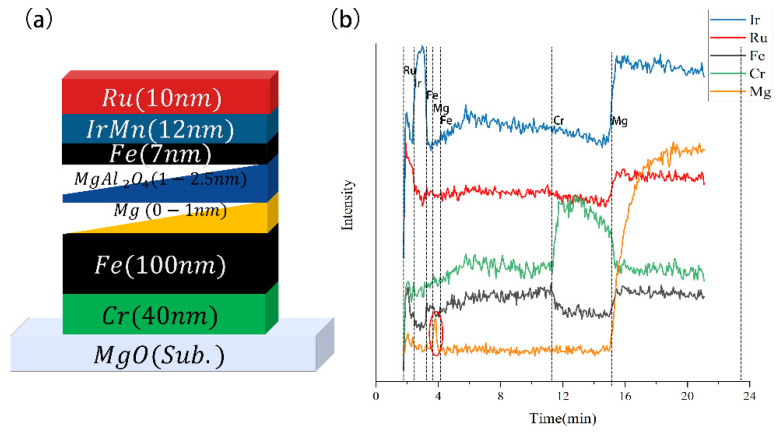
(**a**) The structure of multilayer film sample. (**b**) Spectrum test results of multilayer samples, red circle means the system had a good detection effect for the wedge-shaped thin films of Mg and ${\mathrm{MgAl}}_{2}O_{4}$ as thin as 3.5 nm.

The spectrum test results of multilayer samples are shown in Figure 7; the “main line” intensity of Ir, Ru, Fe, Cr, and Mg changed with time (IrMn used Ir’s detection to represent, MgO and MgAl2O4 used Mg’s detection to represent). According to NIST’s database, we selected the “emission line interval” of Ir as 401 nm–408 nm, the “emission line interval” of Ru as 377 nm–382 nm, the “emission line interval” of Fe as 372 nm–377 nm, the “emission line interval” of Cr as 355 nm–363 nm, and the “emission line interval” of Mg as 283 nm–288 nm. As previously stated, the changes of “main line” intensity demonstrate whether the element was being etched. Ideally, the “main line” of the different elements would appear in the order of film layers over time. In addition, it should be noted that in order to make the contrast obvious, the data have been shifted up and down, which is also the reason why the “Intensity” axis has no unit.

As the results show, it can be found from Figure 7b that the intensity of the “main line” of Ru, Ir, Fe, Mg, Fe, Cr, and Mg elements alternately rose to a high level in the sequence of time. Meanwhile, the division was obvious, which means that the different layers were etched in order, and the boundaries were clear. The acquisition frequency of once every 3 s shows the real-time performance of the etching system was good. In addition, it had a good detection for the wedge-shaped thin films of Mg and ${\mathrm{MgAl}}_{2}O_{4}$, as thin as 3.5 nm in the sample (red circle in the figure). Besides, the Mg layers should be completely removed at the highest point of emission. However, it should be explained that these two Mg layers were wedge-shaped films; the thickness varied from 1 nm to 3.5 nm. Such a thin film could cause some fluctuations in its intensity; besides, the sampling time of 3 s could cause some delays in intensity change. In order to prove that the system can etching end point precisely, we also made a TEM ((Transmission Electron Microscope) test to character the spectral results are consistent with the actual results, as shown in Supplementary Materials. However, this system had its limitations due to the drawbacks of OES. For example, the peaks in the Ru and Ir characteristic intervals in Figure 7 rose when the substrate (MgO) was etched, and the peaks in the Fe characteristic intervals also rose when Ru was etched. This was because the characteristic emission lines of Ru, Ir, and Mg overlapped, and the characteristic emission lines of Fe and Ru also overlapped.

## 5. Conclusions

A system with high precision etching endpoint detecting capability based on optical emission spectroscopy was successfully developed. The design of the system is presented and discussed: an expansion chamber was built on the right side of the etching chamber to fix the optical detector for in-situ detecting, which collected the emission spectrum and transmitted it to the computer so that the endpoint of the etching could be detected by analyzing the spectral data. The position of the optical detector in this system could be adjusted arbitrarily to collect the emission spectrum generated by the sample during the etching process, which makes it superior to other EPDs so far. A feasibility analysis was made by testing simple substances (Al, Cr, Si, and Mg) and multilayer film samples. Meanwhile, a detection limit analysis was also made and the detection limit of the system was 1 nm or thinner. The results indicate that our OES-based etching endpoint detection system has a good real-time performance and excellent endpoint detection capabilities, which is beneficial for fabricating not only spintronic devices but also MEMS or various semiconductor devices.

## Figures and Tables

**Table 1:** Identification of all reported “main lines” in this paper.

Elements	Spectroscopic Notation	Upper Energy Level	Lower Energy Level	Emitted Wavelength
Al	Al I	$3s^{2}3p$	$3s^{2}4s$	396.152
Al	Al III	$2p^{6}5d$	$2p^{6}8f$	398.014
Cu	Cu I	$3d^{10}4s$	$3d^{10}4p$	324.754, 327.395
Mg	Mg I	$2p^{6}3s^{2}$	3s3p	285.2127
Si	Si III	3s3p	3p3d	254.609
Cr	Cr I	$3d^{5}{(}^{6}S)4s$	$3d^{4}{(}^{5}D)4s4p{(}^{3}P^{{}^{\circ}})$	359.350, 360.534
Ir	Ir I	$5d^{8}{(}^{3}F)6s$	$5d^{7}6s{(}^{5}F)6p$	403.376
Ru	Ru I	$4d^{7}(a{\;}^{4}F)5s$	$4d^{7}(a{\;}^{4}F)5p$	372.803
Fe	Fe I	$3d^{6}4s^{2}$	$3d^{6}{(}^{5}D)4s4p{(}^{3}P^{{}^{\circ}})$	374.826

**Table 2:** Intensity under different optical detector angles.

Angles	30°	40°	50°	60°
Average (Intensity)	218.80	262.60	378.10	92.47

**Table 3:** Intensity under different optical detector distances.

Distances	10.8 cm	11.3 cm	12.8 cm	14.3 cm
Average (Intensity)	355.09	321.60	260.18	225.17

## Supplementary Materials

The following are available online at https://www.mdpi.com/article/10.3390/mi13020259/s1.
