# Supplementary material for: An Endpoint Detection System for Ion Beam Etching Using Optical Emission Spectroscopy
Source: Micromachines (Basel). 2022 Feb 5;13(2):259. doi: 10.3390/mi13020259 (PMC8877325; doi:10.3390/mi13020259)
Supplement: Supplementary file 1 [file micromachines-13-00259-s001.zip › micromachines-1581421-supplementary.pdf]

In order to prove that the system can etching end point precisely, we used TEM to analyze the etched samples.

The structure of the multilayer film sample is shown in Fig.S1 (a), which is a typical TMR device, we need to etch to the Ru layer to lead out the bottom electrode. There are two layers of Ru in the whole structure, so its intensity will also increase twice, and the time point of the second increase in intensity is the etching end point. We monitor the variation of Ru characteristic line intensity with time, as shown in Fig.S1 (b), and raise the shutter at the time point of the second increase in intensity. By taking the TEM image of the sample cross section (Fig.S1 (c)), there is a clear conclusion that the sample is indeed etched to the Ru layer in the actual etching process (the thin layer between Ru and SiO_2 is Ta). We have written this part into supplementary materials

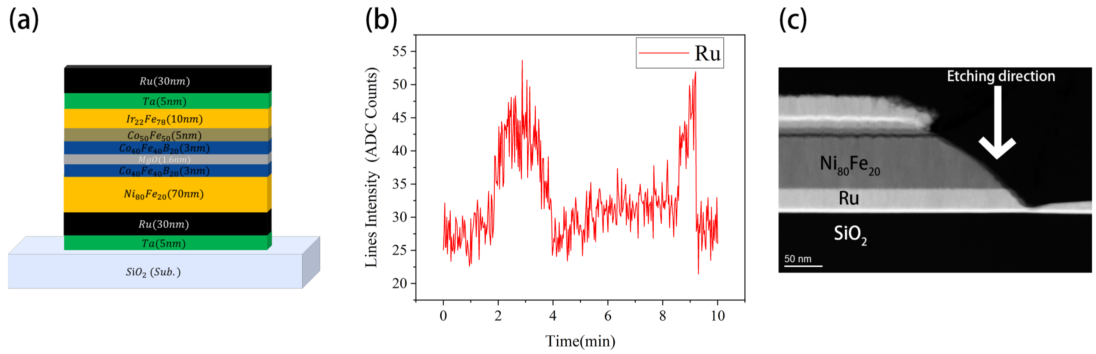

Fig S1. (a) The structure of the multilayer film sample (b) the variation of Ru emission line intensity with time (c) the TEM image of the sample cross section
